# Clinical software for unsupervised automated net water uptake analysis predicts futile recanalization in acute ischemic stroke

**DOI:** 10.3389/fneur.2026.1798635

**Published:** 2026-07-08

**Authors:** Abdallah Aburub, Oussama Dob, Mariana Gurschi, Yashar Aghazadeh, Jumana Jaber, Zaid Al-Tamimi, Zaid Samhan, Lars Timmermann, Christopher Nimsky, Peter Sporns, Gabriel Broocks, André Kemmling

**Affiliations:** 1 Department of Diagnostic and Interventional Neuroradiology, Philipps University of Marburg, Marburg, Germany; 2 Clinic of Diagnostic and Interventional Radiology, Philipps University of Marburg, Marburg, Germany; 3 Department of Neurosurgery, Philipps University of Marburg, Marburg, Germany; 4 Department of Neurology, Philipps University of Marburg, Marburg, Germany; 5 Department of Neuroradiology, University Hospital Basel, Basel, Switzerland; 6 Department of Radiology and Neuroradiology, Stadtspital Zurich Triemli, Zurich, Switzerland; 7 Department of Diagnostic and Interventional Neuroradiology, University Medical Center Hamburg-Eppendorf, Hamburg, Germany; 8 Department of Neuroradiology, HELIOS Kliniken Schwerin, Schwerin, Germany; 9 Department of Interventional Radiology and Neuroradiology, Rhön Klinikum Campus Bad Neustadt, Bad Neustadt an der Saale, Germany

**Keywords:** acute ischemic stroke, automated imaging analysis, cerebral edema, endovascular thrombectomy, futile recanalization, net water uptake, outcome prediction

## Abstract

**Objectives:**

This study aimed to determine whether automated net water uptake (NWU) measured on admission non-contrast computed tomography (CT) independently predicts futile recanalization (FR) in anterior-circulation large vessel occlusion (LVO) stroke treated with endovascular thrombectomy (EVT).

**Methods:**

This was a retrospective single-center cohort study that included consecutive patients presenting at a tertiary care stroke center between January 2023 and April 2025. Patients were included if they presented with an anterior circulation LVO and had successful recanalization (mTICI 2b–3). An automated platform (VEOcore/MRAY) provided the Alberta Stroke Program Early CT Score (ASPECTS), perfusion metrics, and NWU. Associations with FR were analyzed using logistic regression. Discrimination was evaluated using receiver operating characteristic (ROC) analysis and Youden’s J statistic to identify an optimal NWU threshold, reporting the area under the curve (AUC), sensitivity, specificity, and predictive values.

**Results:**

Among 91 patients, 62 (68.1%) achieved recanalization with a 90-day modified Rankin Scale (mRS) score of 0–4, and 29 (31.9%) met the criteria for FR (mRS score 5–6). Those with FR were older (81.6 ± 8.3 vs. 76.9 ± 10.7 years; *p* = 0.044) and had more severe strokes at presentation (median National Institutes of Health Stroke Scale [NIHSS] score of 16 [13–20] vs. 9.5 [6–15]; *p* < 0.001). NWU in the core was higher in FR (21.9% [7.0–29.4]) than in non-FR cases (3.0% [0.5–7.3]; *p* < 0.001) and correlated with 90-day mRS (Pearson *r* = 0.602; 95% CI 0.452–0.719; *p* < 0.001). In multivariable models, NWU remained independently associated with FR (odds ratio [OR] 1.15; 95% CI 1.06–1.24; *p* < 0.001).

**Conclusion:**

Automated NWU on admission CT is an independent, strongly discriminative predictor of FR and 90-day disability. A tiered strategy using >11.5% as a risk flag and >17.5% as a high-specificity rule-in threshold may enhance early triage, prognostication, and clinical trial stratification. Prospective multicenter validation is warranted.

## 1 Introduction

Acute ischemic stroke (AIS) resulting from a large vessel occlusion (LVO) is a primary global cause of mortality and significant long-term disability (1). For eligible patients, endovascular thrombectomy (EVT) has become the definitive standard of care, markedly improving rates of vessel recanalization (2). Despite high rates of technical success with EVT, a considerable number of patients still experience poor clinical outcomes, a challenging phenomenon often described as “futile recanalization (FR)” (3). This discrepancy highlights a critical gap in stroke management: the absence of advanced prognostic biomarkers that can accurately reflect tissue-level injury and reliably predict clinical outcomes beyond the binary assessment of reperfusion success.

Net water uptake (NWU), a quantitative imaging biomarker derived from non-contrast computed tomography (NCCT) at admission, reflects the degree of water accumulation within ischemic brain tissue. Biologically, early ischemia causes a failure of ATP-dependent ion pumps, a loss of transmembrane ionic gradients, intracellular sodium and water influx, and subsequent cytotoxic edema. As tissue water content increases, CT attenuation decreases; NWU therefore estimates the relative reduction in Hounsfield units within the ischemic lesion compared with the anatomically corresponding contralateral hemisphere (4). This CT-density approach has been experimentally and clinically supported by work showing a close relationship between density-derived NWU and direct volumetric measures of lesion water uptake (5).

In LVO stroke treated with thrombectomy, measurable NWU is commonly observed at baseline and varies according to tissue injury severity, infarct age, collateral status, and response to reperfusion. Prior thrombectomy cohorts have reported higher NWU among patients with poor outcomes despite successful recanalization. Nawabi et al. reported mean NWU values of 5.0% in patients with a 90-day modified Rankin Scale (mRS) score of 0–4 compared with 12.1% in those with an mRS score of 5–6 after successful recanalization, with NWU > 10% identifying patients with very poor outcomes (3). More recent LVO–EVT cohorts have reported median baseline NWU values of approximately 6–7%, with significantly higher values among patients with FR. Another successful recanalization cohort found higher NWU in patients with poor 90-day outcomes than in those with favorable outcomes (6, 7). These observations support the concept that NWU functions as a tissue-level marker of ischemic injury rather than a simple surrogate of chronological time. Elevated NWU may indicate accelerated edema formation and irreversible parenchymal injury, helping explain why some patients experience severe disability or death despite technically successful angiographic reperfusion (5). Accordingly, NWU has been associated with malignant cerebral edema, hemorrhagic transformation, poor functional outcome, and FR across acute ischemic stroke studies (8).

Despite this compelling evidence, the widespread clinical adoption of NWU has been hindered by the complexity, time constraints, and operator dependency of manual measurement techniques (9). These approaches often require lesion segmentation supported by CTP maps, which is impractical in time-critical emergency settings (10). Unsupervised learning offers a transformative solution by enabling rapid, standardized, and reproducible NWU quantification; however, to date, NWU quantification has not been evaluated in a real-world stroke triage setting. Therefore, the objective of this study was to evaluate the prognostic value of quantitative NWU measurement via multimodal CT in a real-world clinical setting of acute stroke triage. The study evaluates the prognostic value of NWU quantification for FR using fully automated, unsupervised software during diagnostic imaging in patients with anterior circulation LVO treated with EVT.

## 2 Patients and methods

### 2.1 Study design and setting

This was a retrospective, single-center, observational cohort study conducted at the University Hospital Marburg, a comprehensive stroke center. We included consecutive patients with acute ischemic stroke (AIS) due to an anterior circulation LVO who were treated between January 2023 and April 2025. The study protocol was approved by the local institutional review board (IRB: 25–213 RS), which waived the requirement for written informed consent due to the retrospective and anonymized nature of the analysis. The research was conducted in accordance with the principles of the Declaration of Helsinki.

### 2.2 Study population

Patients were consecutively identified from the local institutional stroke registry according to the following eligibility criteria: (1) acute ischemic stroke due to an LVO in the anterior circulation, confirmed by CT angiography (CTA). Qualifying occlusions included the internal carotid artery terminus (ICA-T), the M1 segment, or a dominant M2 segment of the middle cerebral artery (MCA), defined according to the HERMES Collaboration criteria as an M2 division providing more than 50% of the MCA territory (11); (2) complete admission imaging with a multimodal CT protocol comprising NCCT, CTA, and CT perfusion (CTP); (3) treatment with EVT resulting in successful reperfusion, which was defined as achieving a modified Thrombolysis in Cerebral Infarction (mTICI) score of 2b–3; and (4) available 90-day functional outcome data.

Patients were excluded if they received an intracranial or extracranial stent during the EVT procedure, had a chronic territorial infarction involving more than one-third of the MCA territory in either the ipsilateral or contralateral hemisphere on admission NCCT, or had incomplete or poor-quality imaging (e.g., motion artifacts) that prevented automated analysis. This exclusion criterion was applied qualitatively rather than quantitatively using infarct volume quantification or Alberta Stroke Program Early CT Score (ASPECTS) deduction, because extensive chronic tissue loss could compromise automated NWU measurement through the distortion of either the ischemic lesion region or the contralateral mirrored reference region. The presence of such an infarction was determined by a consensus review of the admission NCCT by two board-certified neuroradiologists blinded to clinical outcomes, with support from prior imaging and clinical history when available.

### 2.3 Variables

All clinical and imaging variables were collected and managed in a secure, anonymized database. The primary outcome was functional status at 90 days, assessed using the mRS. The mRS score was obtained by trained personnel during a scheduled follow-up visit. For analysis, outcomes were dichotomized into the non-FR group, defined as those who achieved a non-poor outcome (mRS score of 0–4) with successful recanalization, and the FR group, defined as those who had a poor outcome (mRS score of 5–6) despite successful recanalization. This definition was chosen to capture the most clinically severe and unequivocal form of FR, in which successful angiographic reperfusion fails to prevent severe disability or death (12–14); in addition, it served to reduce outcome heterogeneity and focus on patients in whom recanalization was least likely to have translated into meaningful clinical benefit. The primary exposure variable was the NWU value, which was automatically quantified from the admission NCCT using the VEOcore software. Potential confounders and other clinically relevant variables were extracted from electronic health records. These included demographic data (age, sex), baseline stroke severity (National Institutes of Health Stroke Scale [NIHSS] score on admission), and key imaging markers derived from the automated analysis, such as ischemic core volume and the ASPECTS.

### 2.4 Data sources and measurement

#### 2.4.1 Imaging acquisition protocol

All patients underwent baseline neuroimaging using a standardized acute stroke imaging protocol on a multi-detector CT scanner (Somatom Definition AS, Siemens Healthineers, Erlangen, Germany). The protocol included NCCT, CT angiography (CTA), and CT perfusion (CTP). NCCT was acquired with a tube voltage of 120 kV, a tube current of 300 mA, a slice thickness of 0.75 mm, and a reconstruction matrix of 512 × 512 using a soft-tissue kernel. CTP imaging was performed at 80 kV and 140 mA with a dynamic acquisition time of 40 s and a temporal resolution of 1.5 s. A weight-adapted contrast bolus (50–70 mL) was administered at a rate of 5 mL/s. The resulting CTP source data were post-processed using the vendor’s proprietary software (Syngo.via MMWP, Siemens Healthineers) to generate perfusion parameter maps, including cerebral blood flow (CBF), cerebral blood volume (CBV), and time-to-maximum (*T*_max_).

#### 2.4.2 Automated NWU quantification and image analysis

All admission imaging data were subsequently analyzed using the VEOcore CT Perfusion imaging platform in MRAY (mbits imaging GmbH, Heidelberg, Germany), which performs fully automated image processing and tissue quantification, including NWU, ASPECTS, ischemic core, and penumbral volumetry. As part of preprocessing, the software used an automated motion-correction module (VEOcore_control_motion) to align the CTP source images. Quality control checks, including correlation curve analysis, were used to ensure proper correction.

VEOcore is a commercially available, CE-labeled software product for automated acute stroke imaging analysis, integrated into the mRay platform (mbits imaging GmbH, Heidelberg, Germany). The software is designed for automated CT/CTP and MRI-based stroke processing, including core and hypoperfusion segmentation, mismatch visualization, motion and bolus quality control, and structured output generation. In the present study, VEOcore was used as implemented in routine clinical workflow at our center; the software was not modified by the investigators, and all automated outputs were reviewed by experienced neuroradiologists before inclusion in the final analysis.

Perfusion analysis included the generation of perfusion parameter maps and the automated calculation of ischemic core and hypoperfusion volumes:

- Ischemic core: rCBF <30% compared to the contralateral hemisphere
- Hypoperfused tissue: *T*_max_ > 6 s
- Mismatch volume (penumbra): hypoperfusion volume minus ischemic core volume

NWU was quantified through an automated process using the same VEOcore platform (Supplementary Figures S1, S2). The software co-registered the ischemic core lesion from the CTP map onto the corresponding NCCT. Within this defined lesion region, the software identified areas of hypoattenuation consistent with early ischemic changes and measured the mean HU density of the ischemic tissue ( ${\mathrm{HU}}_{\text{ischemic}})$ . A mirrored region of interest (ROI) was automatically generated in the contralateral, anatomically corresponding area to determine the mean density of normal tissue ( ${\mathrm{HU}}_{\text{normal}})$ . NWU was then calculated as follows: (15).


$$\mathrm{NWU}=(\frac{{\mathrm{HU}}_{\text{normal}}-{\mathrm{HU}}_{\text{ischemic}}}{{\mathrm{HU}}_{\text{normal}}})\times 100$$

HU histogram values were clamped between 20 and 80 and sampled to exclude the misclassification of voxels containing bone, calcification, or cerebrospinal fluid.

All automated imaging results, including NWU values and ASPECTS, were independently and visually reviewed for accuracy by two board-certified neuroradiologists with over 10 years of experience each (YA and MG). Both reviewers were blinded to all clinical and outcome data. Cases with substantial artifacts or clear software processing errors were excluded from the final analysis.

### 2.5 Statistical methods

Statistical analyses were performed using R (Version 4.3.0; R Foundation for Statistical Computing, Vienna, Austria). Continuous variables were tested for normality using the Shapiro–Wilk test. Normally distributed variables are reported as mean ± standard deviation (SD) and were compared using Student’s *t*-test. Non-normally distributed variables are presented as medians with interquartile ranges (IQRs) and were compared using the Mann–Whitney *U*-test. Categorical variables are reported as frequencies and percentages and were compared using the chi-square or Fisher’s exact test, as appropriate. Binary logistic regression was used to assess the association between automated NWU and FR status. A multivariable model was developed to adjust for clinically relevant confounders, including age, admission NIHSS score, and ASPECTS. Receiver operating characteristic (ROC) analysis was conducted to evaluate the ability of NWU to discriminate between FR and non-FR. The area under the curve (AUC) was calculated, and the optimal cutoff value for NWU was determined using Youden’s J statistic to maximize the sum of sensitivity and specificity. Sensitivity, specificity, and positive and negative predictive values were calculated for this optimal threshold. Exploratory analyses were conducted to evaluate the association between core NWU and outcomes beyond the primary FR definition. Associations with the full 90-day mRS distribution and NIHSS score at discharge were assessed using Spearman correlation. Core NWU was also compared between patients with a favorable functional outcome (mRS score of 0–2) and an unfavorable functional outcome (mRS score of 3–6). The association between core NWU and hemorrhagic transformation was explored using Mann–Whitney *U*-test. A two-sided *p*-value of <0.05 was considered statistically significant.

## 3 Results

### 3.1 Baseline patient characteristics

**Figure 1:**
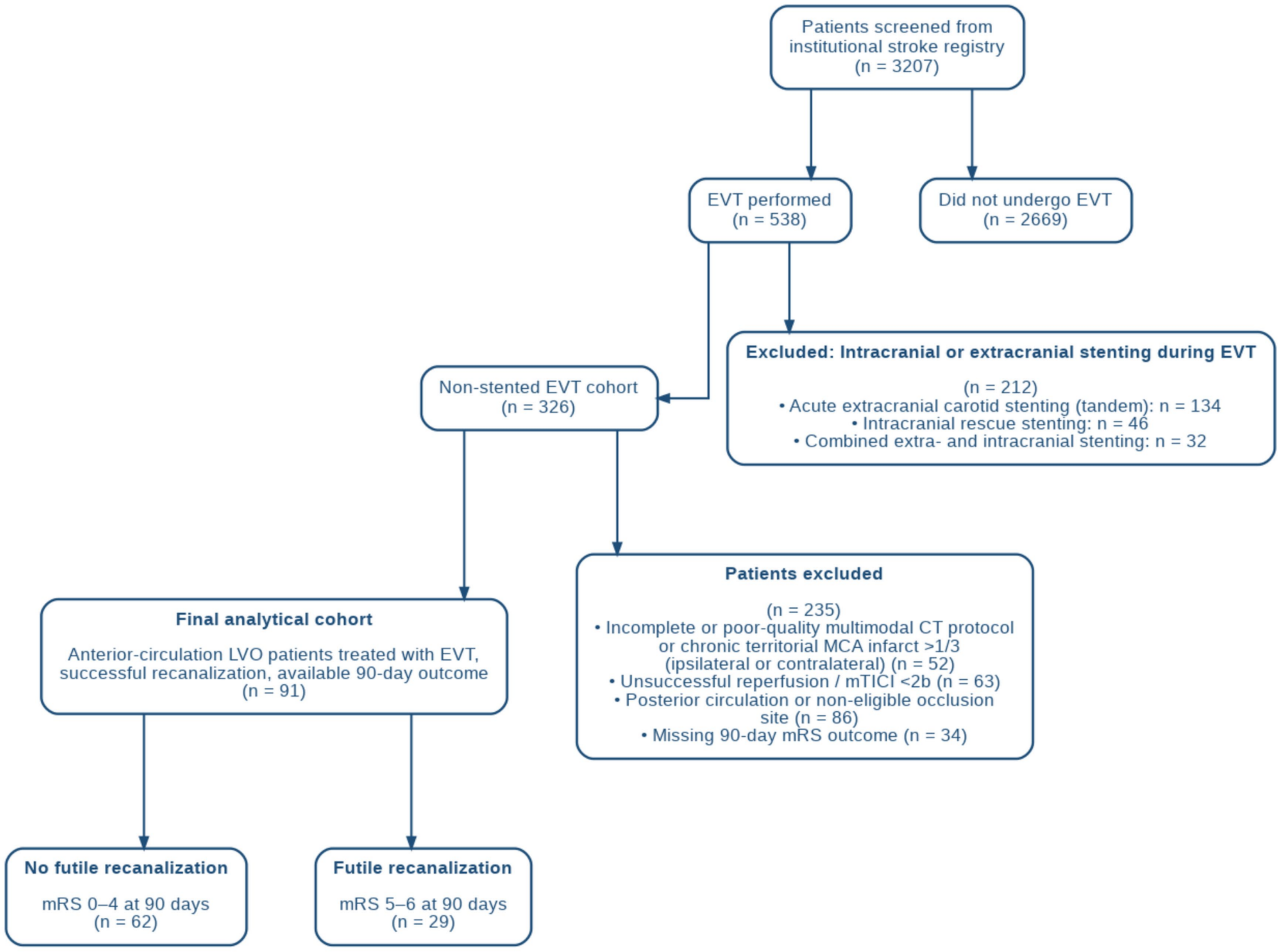
Patient selection flow diagram. This flow diagram shows the number of patients screened, reasons for exclusion, and the final classification of the analytical cohort by futile recanalization status. Imaging-related exclusions included an incomplete or poor-quality multimodal CT scan or a chronic territorial MCA infarction involving more than one-third of the ipsilateral or contralateral MCA territory. EVT, endovascular thrombectomy; FR, futile recanalization; LVO, large vessel occlusion; MCA, middle cerebral artery; mRS, modified Rankin Scale; mTICI, modified thrombolysis in cerebral infarction.

During the study period, 3,207 patients were screened from the institutional stroke registry. Of the 538 patients who underwent EVT, 326 (60.6%) did not receive intracranial or extracranial stenting and were assessed against the remaining eligibility criteria. Patients who received adjunctive stenting during EVT (*n* = 212, 39.4%) were excluded from the subsequent eligibility assessment; these included acute extracranial carotid stenting (*n* = 134), intracranial rescue stenting (*n* = 46), and combined extracranial and intracranial stenting (*n* = 32). This exclusion was applied to maintain a more homogeneous EVT-only cohort and to avoid confounding by tandem–lesion treatment, rescue stenting, procedure-related heterogeneity, and periprocedural antithrombotic management. After excluding patients who did not meet the predefined eligibility criteria, 91 patients were included in the final analytical cohort. Of these, 62 (68.1%) patients were classified as non-FR, and 29 (31.9%) patients met the definition of FR. Patient selection is summarized in Figure 1.

Patients with FR were significantly older than those with non-FR (mean age 81.6 ± 8.3 vs. 76.9 ± 10.7 years; *p* = 0.044). Although the distribution of sex, smoking status, hypertension, diabetes, and coronary heart disease (CHD) did not differ significantly between the two groups, dyslipidemia was notably more common in the non-FR group (93.5%) than the FR group (62.1%; *p* < 0.001). Stroke severity at admission, as measured by the NIHSS score, was significantly higher among patients with FR than among those with non-FR (median, 16 [IQR, 13–20] vs. 9.5 [IQR, 6–15]; *p* < 0.001). The baseline ASPECTS was lower among patients with FR than among those with non-FR (median, 8.0 [IQR, 5.0–9.0] vs. 9.0 [IQR, 8.0–10.0]; *p* = 0.0018). Occlusion-site distribution did not differ significantly between groups (*p* = 0.345), with M1 and M2 occlusions accounting for most cases in both groups, as reported in Table 1.

**Table 1:** Baseline characteristics of patients stratified by futile recanalization status.

Variables		All patients (*n* = 91)	No FR (*n* = 62)	FR (*n* = 29)	*p*-value
Age	Mean ± SD	78.4 ± 10.2	76.9 ± 10.7	81.6 ± 8.3	0.044
Sex	Male	39 (42.9%)	27 (43.5%)	12 (41.4%)	0.846
	Female	52 (57.1%)	35 (56.5%)	17 (58.6%)	
Smoking	No	66 (72.5%)	43 (69.4%)	23 (79.3%)	0.322
	Yes	25 (27.5%)	19 (30.6%)	6 (20.7%)	
Hypertension	No	22 (24.2%)	16 (25.8%)	6 (20.7%)	0.595
	Yes	69 (75.8%)	46 (74.2%)	23 (79.3%)	
Diabetes	No	64 (70.3%)	42 (67.7%)	22 (75.9%)	0.429
	Yes	27 (29.7%)	20 (32.3%)	7 (24.1%)	
CHD	No	48 (52.7%)	30 (48.4%)	18 (62.1%)	0.233
	Yes	43 (47.3%)	32 (51.6%)	11 (37.9%)	
Dyslipidemia	No	15 (16.5%)	4 (6.5%)	11 (37.9%)	<0.001
	Yes	76 (83.5%)	58 (93.5%)	18 (62.1%)	
Peri-medications	None	21 (23.1%)	9 (14.5%)	12 (41.4%)	0.071
	SAPT	35 (38.5%)	28 (45.2%)	7 (24.1%)	
	DAPT	6 (6.6%)	4 (6.5%)	2 (6.9%)	
	DOAC only	17 (18.7%)	14 (22.6%)	3 (10.3%)	
	DOAC + SAPT	7 (7.7%)	3 (4.8%)	4 (13.8%)	
	VKa	3 (3.3%)	2 (3.2%)	1 (3.4%)	
	DOAC + VKa	1 (1.1%)	1 (1.6%)	0 (0.0%)	
	DOAC + SAPT + VKa	1 (1.1%)	1 (1.6%)	0 (0.0%)	
Side	Left	51 (56.0%)	31 (50.0%)	20 (69.0%)	0.089
	Right	40 (44.0%)	31 (50.0%)	9 (31.0%)	
NIHSS at admission	Median (IQR)	11 (6–17)	9.5 (6–15)	16 (13–20)	<0.001
ASPECTS	Median (IQR)	9.0 (8.0–10.0)	9.0 (8.0–10.0)	8.0 (5.0–9.0)	0.0018
Occlusion site	M1	44 (48.4%)	29 (46.8%)	15 (51.7%)	0.345
	M2	39 (42.9%)	28 (45.2%)	11 (37.9%)	
	Carotids-T	5 (5.5%)	2 (3.2%)	3 (10.3%)	
	Others	3 (3.3%)	3 (4.8%)	0 (0.0%)	

ASPECTS: Alberta Stroke Program Early CT Score; NIHSS: National Institutes of Health Stroke Scale; CHD: coronary heart disease; SAPT: single antiplatelet therapy; DAPT: dual antiplatelet therapy; DOAC: direct oral anticoagulant; VKA: vitamin K antagonist. Other occlusion sites included combined M1 + M2 and M1 + ICA-T occlusions.

### 3.2 Clinical and imaging factors associated with futile recanalization

At discharge, patients with FR had a significantly higher median NIHSS score than those with non-FR (10 [IQR: 4–17] vs. 3 [IQR: 0–5]; *p* < 0.001). Representative cases illustrating discordance between ASPECTS, automated NWU, and clinical outcome are provided in Supplementary Figures S1, S2.

Rates of intravenous thrombolysis were comparable between groups (37.9% vs. 43.5%; *p* = 0.613). However, hemorrhagic transformation occurred more frequently among those with FR (44.8% vs. 22.6%; *p* = 0.030), as did malignant infarction (41.4% vs. 1.6%; *p* < 0.001). Imaging analysis showed significantly higher NWU in the ischemic core among patients with FR (median, 21.9% vs. 3%; *p* < 0.001), and a greater proportion had NWU values >11.5% (62.1% vs. 9.7%; *p* < 0.001). While hypoperfusion volumes did not differ significantly, the mismatch ratio was notably lower in the FR group (2.9 vs. 6.1; *p* = 0.008). There were no significant differences in mismatch volume or CBF between the groups. Regarding reperfusion status, successful recanalization (TICI score of 2b–3) was achieved in all patients, though TICI 3 scores were more frequent in the non-FR group (79.0 vs. 65.5%; *p* = 0.301), as shown in Table 2.

**Table 2:** Clinical and imaging characteristics associated with futile recanalization.

Variables		All patients (*n* = 91)	No FR (*n* = 62)	FR (*n* = 29)	*p*-value
NIHSS at discharge	Median (IQR)	3 (2–8)	3 (0–5)	10 (4–17)	<0.001
Intravenous thrombolysis	No	53 (58.2%)	35 (56.5%)	18 (62.1%)	0.613
	Yes	38 (41.8%)	27 (43.5%)	11 (37.9%)	
Hemorrhagic transformation	No	64 (70.3%)	48 (77.4%)	16 (55.2%)	0.030
	Yes	27 (29.7%)	14 (22.6%)	13 (44.8%)	
Malignant Infarction	No	78 (85.7%)	61 (98.4%)	17 (58.6%)	<0.001
	Yes	13 (14.3%)	1 (1.6%)	12 (41.4%)	
NWU core	Median (IQR)	5.5 (1.0–12.5)	3 (0.5–7.3)	21.9 (7.0–29.4)	<0.001
NWU	≤11.5%	67 (73.6%)	56 (90.3%)	11 (37.9%)	<0.001
	>11.5%	24 (26.4%)	6 (9.7%)	18 (62.1%)	
Mismatch Volume	Median (IQR)	77 (40.5–105.5)	77 (44.5–105.5)	71 (37–105.5)	0.969
CBF	Median (IQR)	3 (0–34)	3 (0–23.5)	4 (0–51)	0.584
Mismatch ratio	Median (IQR)	4.2 (2.8–11)	6.1 (3.2–16.9)	2.9 (2.3–3.5)	0.008
Hypoperfusion *T*_max_	Median (IQR)	90 (40–144.5)	85.5 (41.5–141.5)	104 (39–193)	0.291
TICI classification	2b	11 (12.1%)	7 (11.3%)	4 (13.8%)	0.301
	2c	12 (13.2%)	6 (9.7%)	6 (20.7%)	
	3	68 (74.7%)	49 (79.0%)	19 (65.5%)	

NIHSS: National Institutes of Health Stroke Scale; NWU: net water uptake; CBF: cerebral blood flow; *T*_max_: time-to-maximum; TICI: thrombolysis in cerebral infarction classification.

**Figure 2:**
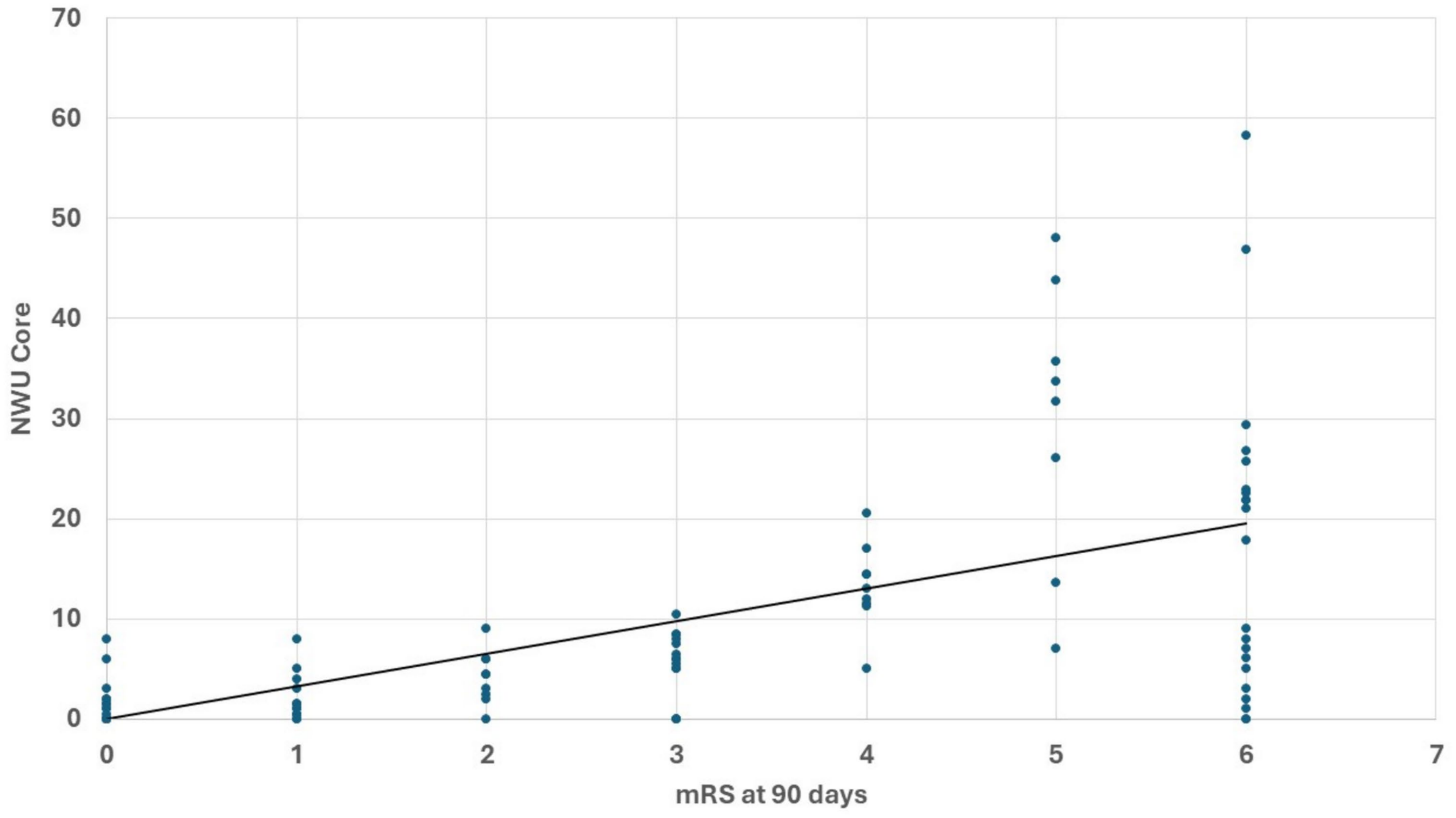
Correlation between core net water uptake (NWU) and 90-day functional outcome (mRS score).

Figure 2 shows that there was a significant positive correlation between NWU in the ischemic core and 90-day mRS scores (Pearson’s *r* = 0.602; 95% CI: 0.452–0.719, *p* < 0.001).

### 3.3 Diagnostic performance of imaging and clinical predictors for FR

**Figure 3:**
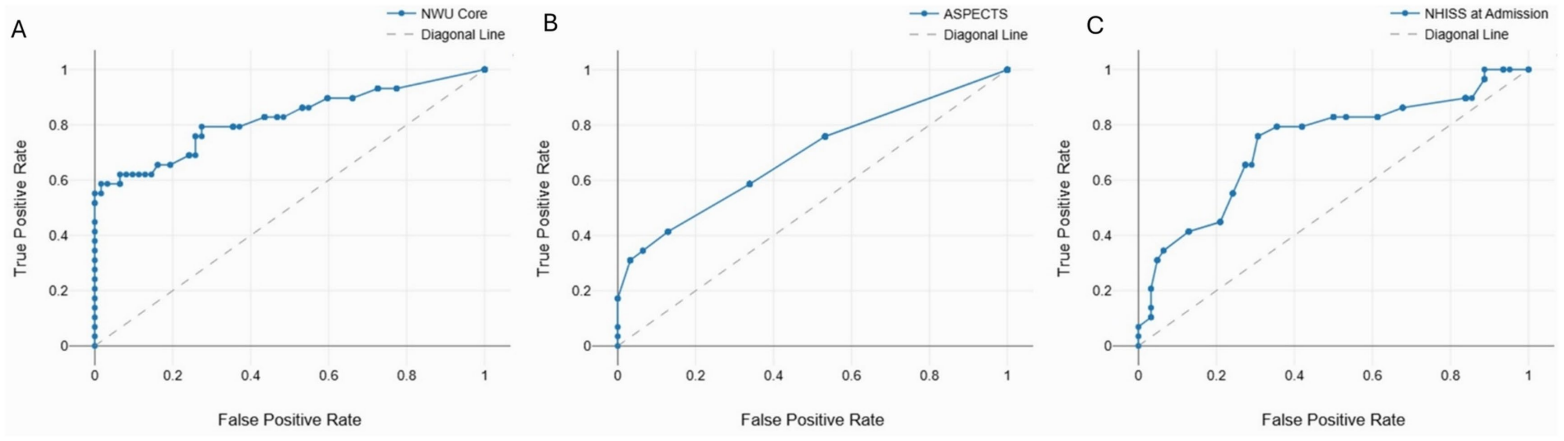
ROC curve analysis of predictors for futile recanalization. **(A)** NWU core, **(B)** ASPECTS, and **(C)** NIHSS at admission in predicting futile recanalization.

Among the evaluated predictors for FR, NWU thresholds demonstrated the strongest diagnostic performance. NWU > 11.5% showed a sensitivity of 62.1% (95% CI: 42.3–79.3), specificity of 90.3% (95% CI: 80.1–96.4), PPV of 75.0% (95% CI: 57.1–89.1), and NPV of 83.6% (95% CI: 76.0–89.1), with an overall accuracy of 81.3%. Increasing the threshold to NWU > 17.5% improved specificity to 98.4% (95% CI: 91.3–100.0) and PPV to 94.4% (95% CI: 70.4–99.2), though sensitivity decreased to 58.6% (95% CI: 38.9–76.5). An ASPECTS score ≤7.5 had a sensitivity of 41.4% (95% CI: 23.5–61.1) and specificity of 87.1% (95% CI: 76.2–94.3), while a mismatch volume >72 mL had lower sensitivity and specificity (51.7 and 58.1%, respectively). Overall, NWU > 17.5% had the highest accuracy (85.7%), as shown in Table 3 and Figure 3.

**Table 3:** Diagnostic accuracy of imaging and clinical predictors for futile recanalization.

Predicting factors	Sensitivity, % (95%CI)	Specificity, % (95%CI)	PPV, % (95% CI)	NPV, % (95% CI)	Accuracy
NWU > 11.5%	62.1 (42.3–79.3)	90.3 (80.1–96.4)	75.0 (57.1–89.1)	83.6 (76.0–89.1)	81.3 (71.8–88.7)
NWU > 17.5	58.6 (38.9–76.5)	98.4 (91.3–100.0)	94.4 (70.4–99.2)	83.6 (76.7–88.7)	85.7 (76.8–92.2)
ASPECTS ≤ 7.5	41.4 (23.5–61.1)	87.1 (76.2–94.3)	60.0 (40.8–76.6)	76.1 (69.8–81.4)	72.5 (62.2–81.4)
Mismatch volume > 72	51.7 (32.5–70.6)	58.1 (44.9–70.5)	36.6 (26.7–47.7)	72.0 (62.5–79.8)	56.0 (45.3–66.4)
CBF Core < 70	24.1 (10.3–43.5)	95.2 (86.5–99.0)	70.0 (39.4–89.3)	72.8 (68.4–76.8)	72.5 (62.2–81.4)
Hypoperfusion *T*_max_ < 192	27.6 (12.7–47.2)	95.2 (86.5–99.0)	72.7 (43.2–90.3)	73.8 (69.0–78.0)	73.6 (63.4–82.3)
Mismatch ratio > 3.9	80.0 (51.9–95.7)	72.2 (54.8–85.8)	54.6 (40.1–68.3)	89.7 (75.5–96.1)	74.5 (60.4–85.7)
NIHSS ≥ 12.5	75.9 (56.5–89.7)	69.4 (56.4–80.4)	53.7 (43.0–64.0)	86.0 (75.9–92.3)	71.4 (61.0–80.4)

NWU: net water uptake; ASPECTS: Alberta Stroke Program Early CT Score; CBF: cerebral blood flow; *T*_max_: time-to-maximum; NIHSS: National Institutes of Health Stroke Scale; CTP: computed tomography perfusion.

### 3.4 Regression analysis and model performance for predicting FR

In univariable logistic regression, dichotomized NWU core thresholds showed a strong association: NWU > 11.5% was associated with 15-fold increased odds of FR (OR: 15.27; 95% CI: 4.95–47.17; *p* < 0.001), while NWU > 17.5% conferred an even higher risk (OR: 86.42; 95% CI: 10.48–712.49; *p* < 0.001), as shown in Table 4.

**Table 4:** Univariable and multivariable logistic regression analyses for predicting futile recanalization.

Predictors	Univariable		Multivariable Model 1		Multivariable Model 2		Multivariable Model 3	
	OR (95% CI)	*p*-value	OR (95% CI)	*p*-value	OR (95% CI)	*p*-value	OR (95% CI)	*p*-value
Age	1.05 (1.00–1.11)	0.049	1.04 (0.99–1.10)	0.120	1.05 (0.98–1.13)	0.141	1.05 (0.99–1.11)	0.123
NIHSS at admission	1.16 (1.07–1.26)	<0.001	1.12 (1.03–1.22)	0.011	1.03 (0.93–1.15)	0.538	1.07 (0.97–1.18)	0.160
ASPECTS	0.64 (0.49–0.83)	<0.001	0.72 (0.54–0.96)	0.024	0.79 (0.57–1.09)	0.152	0.77 (0.56–1.07)	0.115
NWU Core	1.18 (1.09–1.27)	<0.001	–	–	1.15 (1.06–1.24)	<0.001		
NWU core [>11.5]	15.27 (4.95–47.17)	<0.001	–	–	–	–	10.02 (2.80–35.84)	<0.001
NWU core [>17.5]	86.42 (10.48–712.49)	<0.001	–	–	–	–		
Model fitness and performance								
AIC	–		98.23		79.45		86.35	
BIC	–		108.28		92.00		98.90	
*R* ^2^_McF_	–		0.21		0.39		0.33	
*R* ^2^ *_T_*	–		0.26		0.46		0.40	
*X* ^2^	–		23.68		44.46		37.56	
*p*-value	–		<0.001		<0.001		<0.001	
AUC	–		0.79		0.86		0.85	
Sensitivity	–		0.41		0.59		0.62	
Specificity	–		0.94		0.94		0.94	
Accuracy			0.77		0.87		0.84	

Three multivariable models were tested: Model 1 (age, NIHSS at admission, ASPECTS); Model 2 (Model 1 + NWU core as a continuous variable); and Model 3 (Model 1 + NWU dichotomized at >11.5%). Model performance metrics include AIC, BIC, pseudo-*R*^2^ (McFadden’s and Tjur’s), likelihood ratio test (*X*^2^), area under the curve (AUC), sensitivity, specificity, and accuracy. OR: odds ratio; CI: confidence interval; AIC: Akaike information criterion; BIC: Bayesian information criterion.

In the multivariable analysis, Model 1 (clinical model: age, NIHSS score, and ASPECTS) showed that the NIHSS score and ASPECTS remained significant predictors (*p* = 0.011 and *p* = 0.024, respectively), whereas age did not. Incorporating NWU core as a continuous variable (Model 2) improved model performance, with NWU remaining an independent predictor (OR: 1.15; 95% CI: 1.06–1.24; *p* < 0.001). Similarly, adding the dichotomized NWU > 11.5% (Model 3) was strongly associated with FR (OR: 10.02; 95% CI: 2.80–35.84; *p* < 0.001). Model 2 had the best overall performance, with the lowest Akaike Information Criterion (AIC; 79.45), the highest *R*^2^ (McFadden: 0.39, Tjur: 0.46), and an AUC of 0.86. Both Models 2 and 3 showed high specificity (94%) and improved sensitivity (59 and 62%, respectively).

### 3.5 Exploratory analyses of NWU across functional outcomes and hemorrhagic transformation

Core NWU showed a strong positive correlation with the 90-day mRS score (Spearman’s *ρ* = 0.853; *p* < 0.001) and a moderate positive correlation with the NIHSS score at discharge (Spearman’s *ρ* = 0.663; *p* < 0.001). Patients with an unfavorable functional outcome (mRS score of 3–6) had higher core NWU than those with a favorable functional outcome (mRS score of 0–2; median, 14.6% [IQR, 7.9–25.8] vs. 1.0% [IQR, 0.0–2.3]; *p* < 0.001). Core NWU showed high discrimination for an unfavorable functional outcome (AUC, 0.95; 95% CI, 0.90–0.99). For hemorrhagic transformation, the core NWU was higher in patients with hemorrhagic transformation than in those without it (median, 12.1% [IQR, 5.1–21.2] vs. 4.5% [IQR, 0.5–14.5]; *p* = 0.043). However, the discrimination was modest (AUC, 0.63; 95% CI, 0.51–0.76).

## 4 Discussion

This study demonstrates that admission core NWU quantified by a fully automated, unsupervised platform is strongly associated with FR after technically successful EVT in anterior circulation LVO stroke. Patients with FR had markedly higher NWU, and NWU remained independently associated with FR after adjustment for age, admission NIHSS score, and ASPECTS. In diagnostic analyses, NWU thresholds outperformed conventional clinical and imaging markers, supporting NWU as an objective, tissue-level biomarker that helps explain outcome heterogeneity despite reperfusion success.

Our findings showed that patients who experienced FR had a median core NWU of 21.9% than 3.0% in those with non-FR. This 7-fold difference indicates that the extent of early cytotoxic edema captured by NWU is a major determinant of irreversible tissue injury, shaping long-term functional status. The strong positive correlation between core NWU and 90-day mRS scores provides a continuous, quantitative link between the initial ischemic burden and the clinical trajectory. In addition, multivariable logistic regression confirmed the independent predictive power of NWU. When incorporated with age, admission NIHSS score, and ASPECTS (Model 2), NWU as a continuous variable remained highly significant and improved model performance, suggesting added prognostic value beyond traditional clinical scales or semi-quantitative imaging scores. Furthermore, diagnostic accuracy analysis showed that NWU thresholds can stratify risk: NWU > 17.5% demonstrated exceptional specificity and a high positive predictive value for poor outcomes, identifying patients at extremely high risk for FR and malignant infarction (41.4% in the poor outcome cohort vs. 1.6% in the non-poor outcome group).

The exploratory analyses further suggested that the association between NWU and clinical outcomes was not confined to the prespecified FR endpoint. Core NWU was strongly associated with the full 90-day mRS distribution and was higher among patients with an unfavorable functional outcome, supporting its interpretation as a continuous marker of tissue injury severity rather than only a dichotomous predictor of an extremely poor outcome. This finding is consistent with prior LVO cohorts in which automated or ASPECTS-based NWU correlated with the 90-day mRS score and independently predicted functional outcomes, including in patients with low ASPECTS. It is also supported by a recent meta-analysis of 17 studies that included 2,217 patients, in which admission NWU was significantly higher in patients with poor functional outcome, malignant edema/infarction, and intracranial hemorrhage (16). In the present cohort, NWU was higher in patients with hemorrhagic transformation; however, this association was not retained after adjustment, suggesting that NWU may reflect tissue vulnerability and blood–brain barrier injury but may not independently determine hemorrhagic risk when clinical severity, baseline imaging injury, and thrombolysis exposure are considered. Prior studies have reported that core NWU and particularly penumbra NWU were independently associated with symptomatic intracranial hemorrhage after mechanical thrombectomy. However, the strength of this association varied by reperfusion status and imaging compartment (17). Therefore, NWU may be more robust as a global prognostic marker of tissue injury and disability than as a standalone bleeding risk biomarker in EVT-treated cohorts.

The results of this study strongly support the conceptualization of NWU as a “tissue clock,” a measure of the biological severity and progression of ischemic injury that can be critically desynchronized from the chronological time of symptom onset (3). NWU quantifies the net influx of water into brain cells, a direct consequence of energy failure and ion pump dysfunction in severe ischemia (8). A high NWU value on an early scan signifies an accelerated, severe injury cascade. This rapid water accumulation results from cytotoxic edema driven by the failure of ATP-dependent ion pumps, leading to marked intracellular influx of sodium and water (15, 16, 18, 19). Consequently, a high NWU indicates that, irrespective of the time since symptom onset, the brain parenchyma has already sustained severe and likely irreversible injury (20). Our results, therefore, suggest that, even with the restoration of macrovascular flow via EVT, tissue fate is often already determined by the degree of microvascular failure and cellular injury captured by automated NWU measures.

Collateral circulation and treatment workflow times provide important biological context for interpreting NWU. Robust collateral flow may preserve residual perfusion, slow infarct growth, and delay cytotoxic edema, whereas poor collateralization may accelerate tissue hypoxia, ATP-dependent ion pump failure, microvascular dysfunction, and water accumulation within the ischemic lesion. This relationship is supported by prior AIS–LVO thrombectomy data showing that favorable tissue-level collateral status predicted lower ischemic lesion NWU after treatment and that both favorable collateral status and reduced NWU independently predicted good functional outcome (21). Other studies have linked CTA-assessed collateral status and comprehensive collateral blood flow assessment to early edema progression and edema growth after thrombectomy, suggesting that impaired collateral perfusion may promote edema formation even after successful macrovascular reperfusion (22, 23). Treatment delays may further intensify this process by permitting ongoing infarct growth and progressive tissue water uptake before recanalization, consistent with the established time-dependent decline in EVT benefit (24, 25). Accordingly, NWU should not be viewed as a replacement for collateral or workflow assessment, but as a tissue-state biomarker that may integrate the downstream effects of collateral adequacy, ischemia duration, microvascular failure, and edema progression.

The proposed NWU thresholds should also be interpreted in the context of emerging collateral-sensitive perfusion biomarkers. The cerebral blood volume index (CBVI), which reflects the relative preservation of CBV within hypoperfused tissue compared with the contralateral hemisphere, has been proposed as a surrogate of tissue-level collateral compensation. Recent evidence suggests that a lower CBVI is associated with futile recanalization after EVT in anterior circulation LVO, while a higher CBVI appears to reflect better collateral reserve and more favorable outcomes after thrombectomy (26, 27). CBVI may also be particularly relevant for complication-specific prediction, as a low CBVI has been associated with hemorrhagic transformation in medium-vessel occlusion stroke (28). Therefore, NWU and CBVI should be viewed as potentially complementary rather than competing biomarkers: CBVI reflects residual perfusion and collateral reserve, whereas NWU reflects established tissue water uptake and cytotoxic edema. In borderline EVT candidates, patients with a low ASPECTS, or those with distal or medium-vessel occlusions where the risk–benefit balance is less certain, combining NWU with CBVI or other collateral metrics may improve individualized risk stratification. However, prospective validation is required before these thresholds can be used as standalone decision rules.

Our results align with the study by Broocks et al., who first established quantitative lesion water uptake as a predictor of malignant infarction, showing that patients with malignant courses had significantly higher NWU (15). Our study confirms this association in a cohort in which all patients achieved successful reperfusion, demonstrating that an elevated NWU predicts not only malignant edema but also the broader category of FR. Similarly, our findings align with a recent comprehensive meta-analysis by Ghozy et al. (16), which reported that elevated NWU on admission CT is a robust predictor of poor functional outcomes, malignant cerebral edema (MCE), and hemorrhagic transformation. The prognostic value of NWU in our study is consistent with prior work validating this relationship in both anterior circulation strokes (15) and posterior circulation strokes (29). Furthermore, our finding that a high NWU predicts a poor functional outcome independent of treatment success agrees with that of Nawabi et al. (3), who characterized the link between NWU and FR.

While the prognostic value of NWU has been validated in numerous studies (3, 15, 30), its clinical translation has been limited by manual or semi-automated measurement methods that are time-consuming, expertise-dependent, and prone to inter-reader variability in the time-sensitive emergency setting (8, 9, 31). The primary and most critical contribution of our study is the validation of a fully automated, unsupervised platform (VEOcore in mRay), directly addressing the need for standardization and rapid, reproducible quantification highlighted by multiple research groups (8, 16). The mRay platform has also been supported by Brehm et al., who found it adequate for the primary diagnostic review of stroke imaging on mobile devices (32), effectively transforming NWU into a practical real-time biomarker for the hyperacute workflow. Finally, this work aligns with broader efforts to apply unsupervised learning in stroke imaging beyond semi-quantitative metrics such as ASPECTS, providing objective physics-based biomarkers (33), and potentially avoiding the cross-package inconsistencies reported for automated ASPECTS tools (34).

This study also aligns with other research exploring different methods of NWU quantification. For instance, Ou et al. developed a predictive nomogram using ASPECTS-NWU, a method that averages HU values across affected ASPECTS regions (35). While their study used a different mRS cutoff for poor outcome (mRS score of 3–6) and a different calculation method, they similarly concluded that quantitative edema metrics are vital for prognosis. Similarly, Fu et al. proposed an “Image Patch-based NWU” (IP–NWU) that avoids the need for CTP, achieving an AUC of 0.86 in predicting malignant edema (31). These parallel efforts, including the “blindly outlined” method by Xu et al. (36), all converge on the same conclusion: quantifying ischemic water uptake is a powerful prognostic tool.

A pragmatic interpretation of our two NWU cut points is that an NWU > 11.5% functions as a screening or risk flag threshold, whereas an NWU > 17.5% serves as a rule-in threshold for FR. In our cohort, an NWU > 11.5% identified elevated risk with a sensitivity of 62.1% and a specificity of 90.3%, while an NWU > 17.5% accepted lower sensitivity (58.6%) for near-perfect specificity (98.4%) and a PPV of 94.4%, supporting different use cases in triage vs. definitive decision-making. Comparable work in the literature has used similar but not identical thresholds, with variability largely explained by differences in cohort composition (e.g., anterior vs. posterior circulation and baseline severity), outcome definitions (e.g., mRS scores of a 5–6 vs. 3–6; FR vs. general poor outcome), imaging timing and protocols (CT parameters, co-registration), and NWU calculation methods (core-based vs. territory/ASPECTS-averaged vs. patch-based radiomics) (3, 15, 16). Clinically, an NWU > 11.5% may prompt the escalation of monitoring and preparation for complications and inform consent and expectations, whereas an NWU > 17.5% may indicate tissue irreversibility to prioritize neurocritical care, early neurosurgical consultation, and caution in borderline scenarios where the overall benefit is doubtful.

The clinical implications of these findings are substantial: rapid, automated NWU quantification at admission serves as a robust risk stratification tool that can enhance hyperacute patient selection and prognostication. A high NWU should temper clinical optimism even after a successful intervention by supporting the early anticipation of malignant edema or sICH, whereas—even in the presence of a large perfusion deficit—indicates a more resilient tissue profile that justifies aggressive, expedited treatment. NWU may also guide therapeutic decisions and, as proposed by Cheng et al. (8), could serve as a screening biomarker for anti-edema strategies and trials of neuroprotective or anti-edema agents such as glibenclamide (8). A recent secondary analysis of the randomized TENSION trial (ASPECTS 3–5) showed that admission NWU predicted a worse 90-day disability and modified EVT treatment effect, with diminishing efficacy as early edema increased and no clear evidence of benefit when NWU was ≥15%, reinforcing that tissue vulnerability extends beyond lesion extent alone (37). Furthermore, in patients with a low ASPECTS, a low NWU may delineate those still likely to benefit, as suggested by Broocks et al. (15), and incorporating automated NWU into trial enrolment and stratification can reduce confounding by FR and support its use as a quantitative surrogate endpoint.

The integration of automated NWU into a comprehensive, AI-driven platform represents a logical next step, aligning with radiomics approaches that combine IP–NWU with other imaging features to maximize discriminative performance (31). Future research must prioritize prospective, multicenter validation to confirm the generalizability of these thresholds across diverse populations, scanners, and treatment protocols (16, 35). Concurrently, the standardization and harmonization of NWU computation are essential to address cross-platform variability (9). Finally, combining automated NWU with complementary biomarkers, such as tissue-level collateral metrics (e.g., hypoperfusion intensity ratio [HIR]) and venous outflow profiles (21), may enable the development of robust multimodal prognostic models designed to refine patient selection, guide individualized therapy, and improve outcome prediction at scale.

## 5 Limitations

Several limitations of this study must be acknowledged. First, the retrospective, single-center design may introduce selection bias and limit the generalizability of our findings. Second, the sample size of 91 patients, while sufficient for our primary analysis, is relatively modest. This is particularly relevant for subgroup analyses, and larger cohorts are needed to confirm the stability of the identified NWU thresholds. Third, our definition of a non-poor outcome (mRS score of 0–4) is more inclusive than the traditional definition (mRS score of 0–2). We chose this to align with the concept of FR, where the most devastating outcomes (mRS 5–6: severe disability or death) are of primary interest. Fourth, despite the use of a fully automated platform, potential inaccuracies can still arise from imaging artifacts (e.g., patient motion) or inherent limitations in the CTP-to-NCCT co-registration process, although we sought to mitigate this through rigorous quality control and visual review by experienced neuroradiologists. Fifth, this was a prognostic clinical evaluation, not an independent technical validation of the VEOcore NWU algorithm against manual segmentation, phantom data, or another reference standard. External validation across centers, scanners, acquisition protocols, and software versions is needed before the proposed thresholds can be generalized. Finally, collateralization status, door-to-angiographic reperfusion time, and specialized collateral biomarkers such as CBVI were not collected or extracted in a sufficiently standardized manner for inclusion. Future prospective studies should incorporate standardized collateral assessment, treatment-time metrics, CBVI, and complication-specific endpoints such as hemorrhagic transformation.

## 6 Conclusion

In conclusion, this study demonstrates that quantitative NWU, measured by a fully automated and unsupervised software, is a powerful and independent predictor of FR in patients with AIS undergoing EVT. Operationally, we propose a tiered strategy in which an NWU threshold of >11.5% serves as a practical risk flag, while an NWU threshold of >17.5% functions as a high-specificity rule-in marker for severely injured, low-salvage tissue. By providing a rapid, objective measure of the “tissue clock,” automated NWU quantification has the potential to revolutionize acute stroke prognostication, refine patient selection for advanced therapies, and guide the development of novel neuroprotective strategies. As this technology becomes increasingly integrated into clinical workflows, it promises to advance the field beyond simple recanalization success toward a more nuanced, tissue-based approach to improving patient outcomes.

## Data Availability

The data analyzed in this study is subject to the following licenses/restrictions: The datasets analyzed during the current study are not publicly available due to patient privacy regulations and institutional data protection policies. Requests to access these datasets should be directed to AA – Corresponding Author – abdullahaburub8@gmail.com.
