# Supplementary material for: Clinical software for unsupervised automated net water uptake analysis predicts futile recanalization in acute ischemic stroke
Source: Front Neurol. 2026 Jul 8;17:1798635. doi: 10.3389/fneur.2026.1798635 (PMC13388131; doi:10.3389/fneur.2026.1798635)
Supplement: Supplementary file 1 [file Supplementary_File_1.docx]

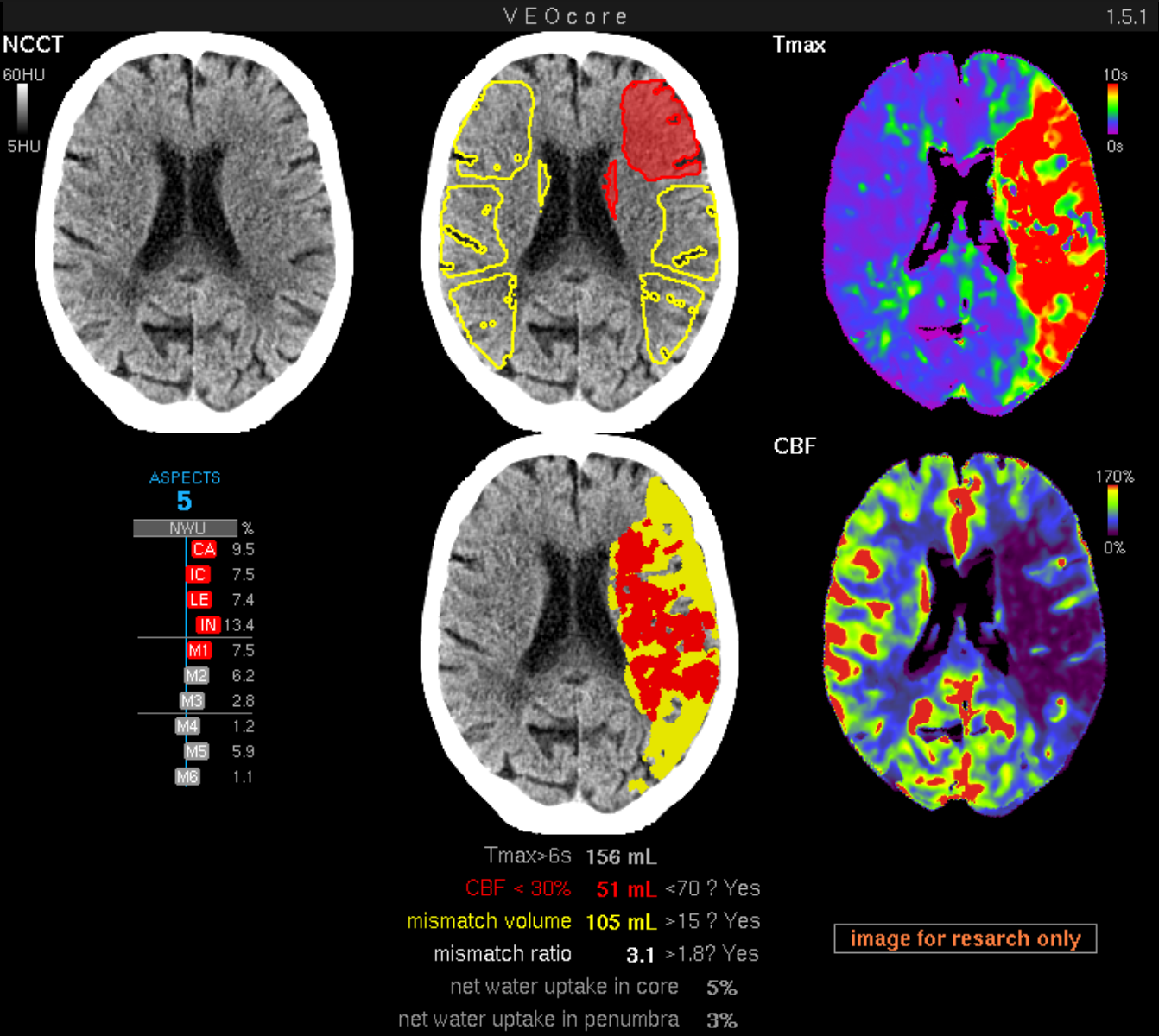


**Supplementary Figure S1:** Representative case with low ASPECTS but low automated net water uptake. Admission NCCT shows extensive early ischemic changes (ASPECTS 5). Automated analysis revealed a large ischemic core (CBF <30%: 51 mL) and hypoperfused volume (Tmax >6 s: 156 mL), but low NWU (5%). Despite the unfavorable ASPECTS, successful reperfusion resulted in a favorable 90-day outcome (mRS 3).


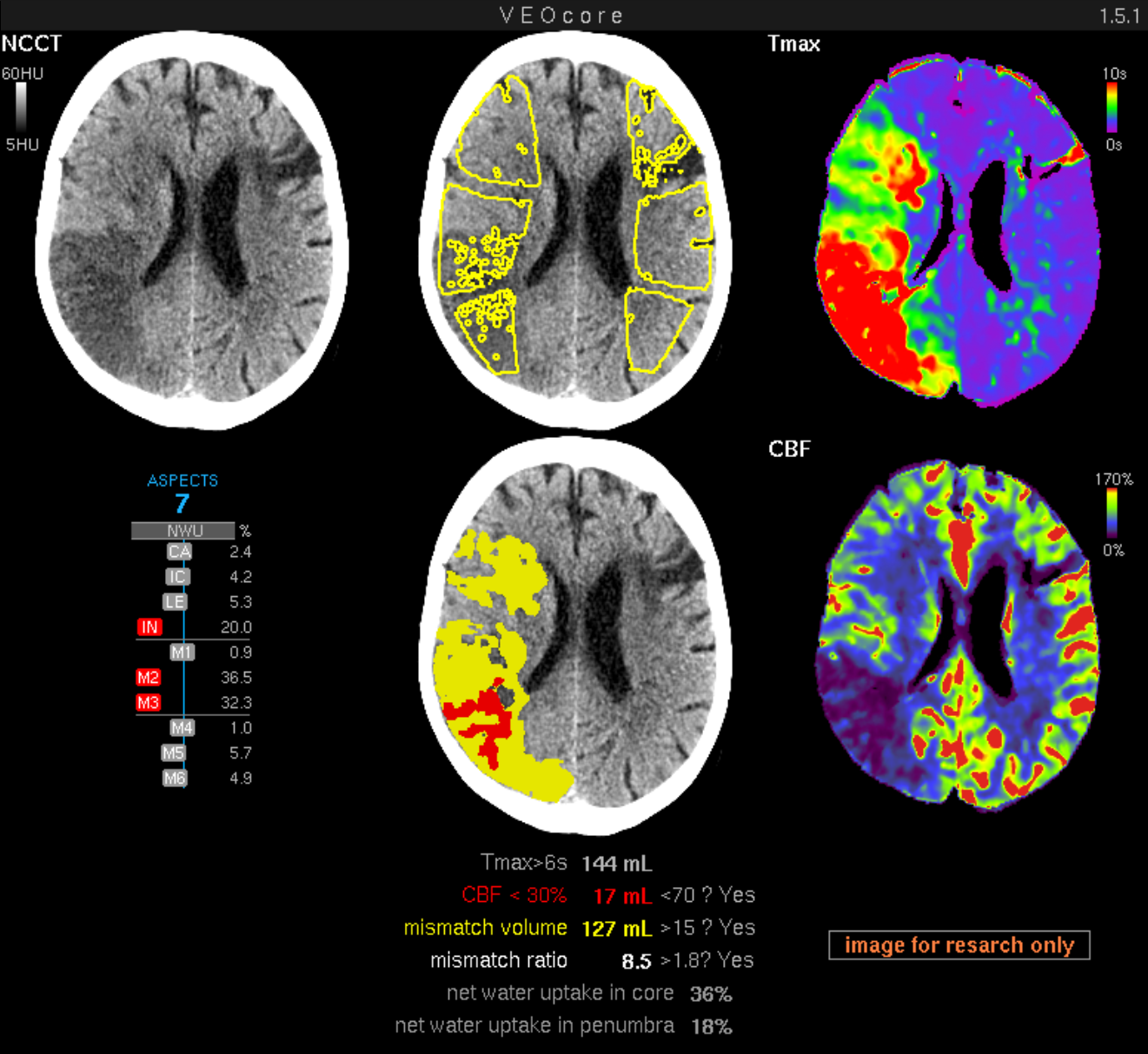


**Supplementary Figure S2:** Representative case with preserved ASPECTS but markedly elevated automated net water uptake. Admission NCCT showed an ASPECTS of 7. Automated perfusion analysis demonstrated a small ischemic core (CBF <30%: 17 mL) with a large hypoperfused volume (Tmax >6 s: 144 mL), resulting in a substantial mismatch volume of 127 mL (mismatch ratio 8.5). Net water uptake was markedly increased in the ischemic core (36%) and penumbra (18%). Despite successful reperfusion, the patient developed a poor functional outcome at 90 days (mRS 5).
